# Editorial: Emerging Therapeutic Approaches for Cystic Fibrosis

**DOI:** 10.3389/fphar.2019.01440

**Published:** 2019-11-29

**Authors:** Miquéias Lopes-Pacheco, Nicoletta Pedemonte, Anthony Kicic

**Affiliations:** ^1^Biosystems & Integrative Sciences Institute (BioISI), Faculty of Sciences, University of Lisbon, Lisbon, Portugal; ^2^UOC Genetica Medica, IRCCS Istituto Giannina Gaslini, Genova, Italy; ^3^Telethon Kids Institute, University of Western Australia, Nedlands, WA, Australia; ^4^Occupation and Environment, School of Public Health, Curtin University, Bentley, WA, Australia; ^5^Department of Respiratory Medicine, Princess Margaret Hospital for Children, Perth, WA, Australia; ^6^Centre for Cell Therapy and Regenerative Medicine, School of Medicine and Pharmacology, The University of Western Australia and Harry Perkins Institute of Medical Research, Nedlands, WA, Australia

**Keywords:** cystic fibrosis transmembrane conductance regulator, mutations, therapeutic approach, drug development, lung, inflammation, infection, modulators

Cystic fibrosis (CF) is the most common life-shortening inherited disease in Caucasian populations. It affects 90,000 to 100,000 individuals worldwide and results in multi-organ dysfunction, although the respiratory disorder represents the major cause of morbidity and mortality of these patients. Understanding of cellular and molecular defects of CF-causing mutations has substantially improved since the discovery of the CF transmembrane conductance regulator (CFTR) gene in 1989 (Kerem et al., 1989; Riordan et al., 1989; Rommens et al., 1989), bringing perspectives for the development of novel therapies. Up to 2012, therapeutic regimens were exclusively focused on disease symptoms. These comprised of physical and inhaled therapies, and numerous daily medications to enhance airway clearance, mitigate inflammation, eradicate lung infections, and supplement absent pancreatic enzymes. Together with early diagnosis and multidisciplinary healthcare, patients’ life expectancy has risen from early childhood in the 1960s to 40 to 50 years in several countries nowadays (Lopes-Pacheco, 2016).

From 2012 to date, four drugs targeting the root cause of CF have reached the market: ivacaftor (Kalydeco^™^), the dual combinations lumacaftor/ivacaftor (Orkambi^™^) and tezacaftor/ivacaftor (Symdeko^™^), and the triple combination elexacaftor/tezacaftor/ivacaftor (Trikafta^™^). In patients carrying specific CF genotypes, these pharmacotherapies have been demonstrating short- and long-term therapeutic outcomes, including improvements in lung function and body mass index, and reduction in detection of *Pseudomonas aeruginosa* and frequency of pulmonary exacerbations (Ramsey et al., 2011; Wainwright et al., 2015; Rowe et al., 2017; Taylor-Cousar et al., 2017; Keating et al., 2018). Other novel therapies targeting CFTR dysfunction, modulating alternative ion channels, and acting on diseases symptoms are under experimental and clinical investigations. Furthermore, new models have been developed to accelerate and continue expanding the pipeline of therapeutic options.

This Research Topic gathers a collection of 22 original and review articles that provide novel information regarding the “Emerging Therapeutic Approaches for Cystic Fibrosis” at basic, translational, and clinical levels. The articles have been divided in three main chapters.

The first chapter is composed by an overview of the literature with some insights on hot topics in CF research, and articles focused on the regulation of CFTR trafficking, stability, and degradation. Pranke et al. introduce the pipeline of emerging therapeutic approaches to restore CFTR function that is being investigated in both experimental and clinical studies. They describe strategies to target specific defects caused by CFTR mutations, including the CFTR modulators (read-through agents, correctors, potentiators, stabilizers, and amplifiers) and antisense oligonucleotides. They also mention the advances in mutation-agnostic approaches, such as cell-based and gene-based therapies, which could be applied for all individuals with CF.

CFTR folding, processing, and trafficking involve complex and hierarchical steps that take place in multiple cellular compartments and engage several networks of protein machineries. Bodas and Vij summarize some key cellular mechanisms that are impaired in CF epithelia, such as protein homeostasis (proteostasis) an autophagy. They also describe some molecules that might modulate the underlying dysfunction in these mechanisms and then control the pathogenesis in CF lung disease. In the review of Cruz et al., the authors elucidate the role of Lemur tyrosine kinase 2 (LMTK2), an incompletely characterized serine-threonine kinase that functions as a multifunctional adaptor. LMTK2 may be a potential biomarker and/or therapeutic target, as it interacts with CFTR and is involved in the regulation of several intracellular events, including protein trafficking, apoptosis, and chloride transport. In the following article, Pesce et al. demonstrate that spautin-1, an autophagy inhibitor, induces a downregulation of the expression and function of F508del-CFTR rescued either by low temperature incubation or by the corrector lumacaftor. This “anti-corrector” compound may also be interesting to investigate novel biological pathways involved in F508del-CFTR degradation.

The CFTR located at the plasma membrane must be a stable protein; otherwise, it will be removed by peripheral quality control mechanisms. Fukuda and Okiyoneda summarize key environmental stresses that may affect CFTR stability at the plasma membrane. They also describe cellular machineries that may be targeted to reduce internalization and deliver to lysosome degradation or to enhance recycling back to the plasma membrane. Loureiro et al. use network biology tools to identify novel candidate proteins involved in CFTR trafficking and membrane stability. They identify GABARAP, NOS2, and SMURF1 as novel regulators of CFTR levels at the plasma membrane. Furthermore, GABARAP knockdown demonstrate to increase the plasma membrane stability of lumacaftor-rescued F508del-CFTR.

The second chapter is dedicated to new methods in CF research and approaches that potentiate CFTR channel or circumvent its dysfunction. A review by Awatade et al. nicely summarize the latest updates in the development of primary epithelial cellular model systems, discussing conventional two-dimensional airway epithelial cell (AEC) cultures, and three dimensional airway and intestinal organoid models and evaluating the limitations and potential improvements in each system, focusing on their application in CF, such as for preclinical pharmacotherapy screening to identify responsive patients. Gianotti et al. describe different methods to expand and differentiate isolated bronchial and nasal cells into fully polarized monolayers of airway epithelium, in order to provide an optimized protocol to support physiopathology analysis and to evaluate therapeutic strategies. A quantitative evaluation by Matthes et al. finally provide evidence for patient-to-patient variation in cell culture responses to correctors that should be carefully considered when CFTR corrector drugs are compared *in vitro* for precision medicine. A group of researchers from Galapagos NV, headed by Conrath (Gees et al.), describes the discovery of two novel CFTR potentiators, named GLPG1837 and GLPG2451, able to improve channel activity on a series of Class III, IV CFTR mutants, and showing no negative interaction with pharmacological rescue of F508del-CFTR trafficking defect. These novel compounds may offer new therapeutic options for CF patients.

Among the mutation-agnostic approaches proposed to overcome the basic defect in CF, two of them are based on the exploitation of alternative targets. Balázs and Mall summarize recent evidences that identified the alternative chloride channel SLC26A9 as a disease modifier and supported its role in the pathophysiology of CF and other chronic lung diseases. Pharmacological activation of SLC26A9 may help to augment the effect of CFTR modulator therapies. Interestingly, Corvol et al. report that SLC26A9 variants, although not associated with lung function variability in untreated patients, are indeed associated with variability in ivacaftor-lung response, suggesting that pharmacogenomics, in addition to personalized medicine, will soon be part of CF patient care.

A review by Kunzelmann et al. is focused on another alternative target that could be exploited for CF therapy, the calcium-dependent chloride channel TMEM16A, which is upregulated during inflammatory lung disease. TMEM16A activation would improve hydration of the airway mucus and increase mucociliary clearance. However, recent evidence suggests that TMEM16A is essential for mucus secretion and possibly also for mucus production, and appears to maintain excessive mucus secretion during inflammatory airway disease. Thus, the still open question is whether TMEM16A needs to be activated or inhibited in CF.

Finally, the last article of this section, by Cossu et al., is focused on an innovative therapeutic approach for the treatment of CF using anionophores, small molecules that facilitate the transmembrane transport of anions. The authors characterized the anion transport mechanism of a synthetic molecule based on the structure of prodigiosine, a red pigment produced by bacteria. This prodigiosin derived ionophore induces anion transport in living cells and displays low toxicity and capacity to transport chloride and bicarbonate, thus constituting a promising starting point for the development of drug candidates for CF therapy.

The third chapter is comprised of those articles that introduce novel therapies that address the causal nature of CF and those tackle the downstream consequences, including infection and inflammation. Donnelley and Parsons initially revisit a long-held curative strategy for CF, namely gene therapy. They review progress in the field made to date, discuss the issues preventing translation into large-scale clinical trials, and outline recent technological advancements, including those made by their team that may overcome these hurdles and see the first trials conducted in the near future. Bardin et al. also reviewed the critical role microRNA (miRNA) play in regulating proteins including CFTR. Specifically, they discuss different strategies to identify dysregulated miRNA in CF and review the potential of antisense techniques that hold considerable potential as future corrective forms of therapy. Finally, Berical et al. discuss the potential of cell-based therapies for CF. In principal, such therapies involve replacing cells that carry a mutant *CFTR* sequence with other cells that express a normal copy of the gene. The authors explore two cell-based therapies, i.e. induced pluripotent stem cells and human bronchial epithelial cell transplantation and how these may address the challenges which currently limits this approach.

In an original research article, Delpiano et al. explore novel approaches to address altered air surface liquid (ASL) pH that occurs in CF. Initially, they trialed inhibiting a H^+^/K^+^-ATPase, namely, ATP12A, *via* ouabain *in vitro* using human AECs grown at air-liquid interface. Although pH was found to be increased, chronic exposure of primary AEC to ouabain, was deleterious on barrier integrity and function. As an alternative approach, the authors then inhibited a related H^+^/K^+^-ATPase, i.e. ATP4A, with the proton pump inhibitor (PPI), esomeprazole. Encouragingly, treatment raised pH without effect on airway barrier integrity or function, illustrating the potential of PPI in addressing altered ASL pH that manifests in CF.

A second group of articles focused on some of the accompanying complications of CF particularly relating to infection and inflammation. Firstly, Ling et al. address early life triggers of inflammation in very young children with CF. In addition to reviewing the types of respiratory infections that occur, the authors focus on viral triggers of inflammation, in particular rhinovirus. In addressing the limitations with currently available therapeutics, they discuss the potential use of “omics” platforms, particularly transcriptomics to elucidate host-pathogen responses to potential identify targets for potential therapeutic intervention. In an original research article, Trend et al. address the issue of antibiotic resistant bacterial lung infections in CF and explore bacteriophages or “viruses that infected specific bacteria” as an alternative therapeutic regimen. Utilizing an *in vitro* airway model comprised of primary AECs from non-CF and CF children, the authors screen candidate phage and identify one, namely E79, to have high specificity and activity against *P. aeruginosa*. It also fails to induce any deleterious effects, including programmed cell death or inflammation in AECs. The findings provide rationale for the continued exploration of bacteriophage therapy to address the global issue of antibiotic resistant bacterial lung infections not only in CF but beyond.

Any new interventional therapy requires a translational pipeline, and Semaniakou et al. review the current animal models of CF available. They discuss the pros and cons to the various models and conclude that presently no one model mirrors the complexity of CF that manifests in humans. However, the authors advocate that these models do have utility and may complement each other to advance our knowledge of CF disease pathogenesis and progression. Finally, Schultz et al. discuss the need of an adaptive trial platform over traditional clinical trial assessment for future therapeutics in CF. They explain the concepts of the Bayesian adaptive platform, where modeling and response adaptive randomization, can facilitate multiple treatments across different management domains and document improvement in patient outcomes throughout the trial period, and outline the necessary steps needed to implement this into practice in the CF sphere.

In conclusion, this issue collates current and emerging therapeutic approaches for CF. These range from; curative strategies, those aimed at correcting defective function, and approaches that target accompanying complications, such as infection and inflammation. The impact of all these approaches if successful will significantly improve not only quality of life of individuals with CF but also extend current life expectancy. With the breath of work currently being conducted by global researchers in their respective fields of specialty, the horizon looks optimistic for several approaches to translate into clinic applications.

## Author Contributions

ML-P, NP, and AK wrote and reviewed the Editorial.

## Conflict of Interest

The authors declare that the research was conducted in the absence of any commercial or financial relationships that could be construed as a potential conflict of interest.
